# And… cut! – how conformational regulation of CRISPR-Cas effectors directs nuclease activity

**DOI:** 10.1042/BCJ20240481

**Published:** 2025-09-25

**Authors:** Roland W. Calvert, Gavin J. Knott

**Affiliations:** Monash Biomedicine Discovery Institute, Department of Biochemistry and Molecular Biology, Biomedicine Discovery Institute, Monash University, Clayton, Victoria, 3800, Australia

**Keywords:** biotechnology, CRISPR, protein conformation, RNA

## Abstract

Controlling the conformation of dynamic protein, RNA and DNA molecules underpins many biological processes, from the activation of enzymes and induction of signalling cascades to cellular replication. Clustered regularly interspaced short palindromic repeats (CRISPR)-associated (Cas) effectors are enzymes tightly controlled by conformational steps that gate activation of nuclease domains core to their function in bacterial adaptive immunity. These precise conformational checkpoints combined with programmable activation specified by RNA guides have driven the success of CRISPR-Cas tools in biotechnology, medicine and beyond. To illustrate the importance of conformation in controlling CRISPR-Cas activity, we review the discrete conformational checkpoints at play in class 2 CRISPR-Cas systems. Using Cas9, Cas12a and Cas13a as examples, we describe how protein and nucleic acid conformations precisely control the loading of guide RNA, the selection of target nucleic acids and the activation of nuclease domains. Much like a director controls the timing of transitions between scenes in a movie, CRISPR effectors use conformational checkpoints to precisely direct their enzymatic activity.

## Conformational checkpoints regulate enzyme activity

Across all domains of life, biological functions are maintained through the intricate and co-ordinated effort of macromolecules. It is the accumulation of minute molecular events that brings about larger observable traits in organisms. As a result, there is a fundamental selective pressure to ensure efficient regulation of biological pathways and to mitigate detrimental biochemical activity. This maintenance is carried out through the intrinsic dynamics of macromolecules like proteins and nucleic acids across a range of conformational states that dictate function.

Clustered regularly interspaced short palindromic repeats (CRISPR) and CRISPR-associated (Cas) effectors are examples of biological systems rich in conformational control. Cas effectors are a vast set of microbial immune ribonucleoprotein complexes that sequence specifically recognise nucleic acids using a guide RNA (gRNA). The gRNA consists of a spacer (an encoded sequence originating from a mobile genetic element) [1–4] and a direct repeat that acts as a handle for Cas protein binding to form effector complexes [5–7]. Once a gRNA spacer base pairs to complementary sequences (hereafter referred to as targets), the Cas-gRNA complex undergoes conformational changes into the Cas-gRNA-target complex. Target-bound effectors have active nuclease domains to eliminate the phage threat by cleaving the target nucleic acid directly or initiating other defence mechanisms, including signalling networks [4,7–10]. In practice, this is a complicated series of events where different domains within the multidomain, or multicomponent effectors, carry out different functions to accommodate target search, recognition and/or interference [11].

CRISPR-Cas effectors are categorised into classes and types based on their overall architecture and phylogeny. Class 1 CRISPR-Cas systems (types I, III and IV) are defined by effector modules comprised of multiple Cas proteins that associate to form the effector complex [12]. In contrast, class 2 CRISPR-Cas systems (types II, V and VI) are defined by a single Cas protein that contains many individual domains responsible for discrete activities in the effector complex [13,14]. Class 2 CRISPR-Cas effectors (namely Cas9, Cas12 and Cas13) have been widely adopted by the scientific community due to their programmable RNA-guided recognition of target nucleic acids [15]. In this context, ‘programmable’ refers to the fact that any string of approximately 20 nucleotides can be encoded into a gRNA for targeting a nucleic acid of interest. Although there are physical constraints that limit the real set of nucleic acids that can be targeted, this programmability has provided a platform to investigate important biological questions and develop techniques to predictably manipulate nucleic acids such as gene editing, transcriptome engineering and nucleic acid diagnostics [16,17]. Just as with any tool, however, a thorough understanding of its mechanisms and limitations elucidates best practices and possible improvements. Failure to account for these may drastically and negatively affect experimental outcomes, especially as CRISPR-Cas technology is adapted for therapeutic and agricultural purposes [18,19].

In this review, we highlight the key molecular transitions that CRISPR-Cas effectors undergo to recognise targets and initiate nuclease activity. While class 1 systems are applied as biotechnologies [20–22], we focus on the class 2 CRISPR-Cas systems that have proven advantageous for biotechnology [16] (Table 1). We also explore the relationship between conformational control and evolution. By showcasing the conformational mechanisms of CRISPR-Cas effectors, we highlight how our understanding of these systems enables their application to biotechnology and sets the stage for further optimisation of existing practices in the field.

**Table 1:** Structural and functional characteristics of Cas9, Cas12a and Cas13a effectors

	Cas9	Cas12a	Cas13a
(s)gRNA size	~100 nt	~40 nt	~50 nt
Spacer position	5′ spacer	3′ spacer	3′ spacer
Processing nuclease	No	Yes	Yes
PAM requirement	Yes	Yes	No
Nuclease domain(s)	HNH & RuvC	RuvC	HEPN
Target	dsDNA	dsDNA/ssDNA	ssRNA
Target (*cis*-) cleavage	dsDNA	dsDNA/ssDNA	ssRNA
*Trans*-cleavage	No	ssDNA	ssRNA

## Directing the cut by CRISPR-Cas9

**Figure 1:**
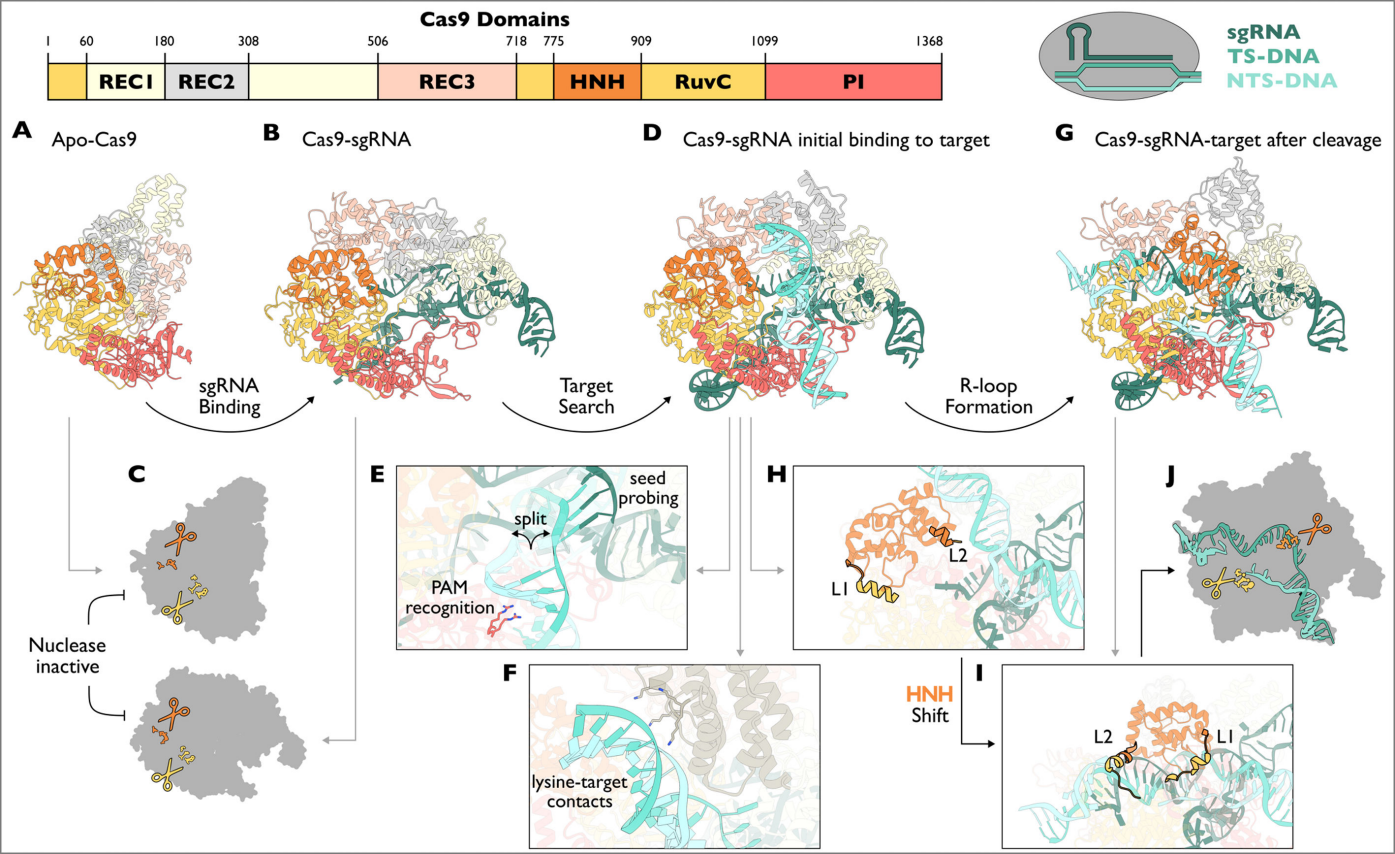
Conformational checkpoints and molecular mechanisms of *Streptococcus* *pyogenes* Cas9. **(A**) Apo-SpCas9 poised to bind sgRNA. (PDB: 4CMQ) (**B**) SpCas9-sgRNA with REC domains rotated from (**A**) to now bind sgRNA. sgRNA is long enough to interact with REC, PI and RuvC domains. (PDB: 4ZT0) (**C**) HNH and RuvC nucleases are in inactive conformations in apo-SpCas9 or SpCas9-sgRNA complexes. (**D**) SpCas9-sgRNA binding target with PAM and 3 bp complementarity to sgRNA spacer. (PDB: 7S38) (**E**) PAM-interacting residues make specific contacts with the NGG sequence on NTS to facilitate DNA unwinding and to allow sgRNA seed to base pair with the TS. PAM recognition of arginine residues – Arg1333 and Arg1335. (**F**) REC2 lysine residues interact with the target duplex. REC2 duplex-binding lysine residues – Lys233, Lys234, Lys253 and Lys263. (**G**) Post-cleavage SpCas9-sgRNA-target with full sgRNA-target complementarity. (PDB: 7S4X) (**H**) HNH domain of structure in (**D**) positioned by linkers L1 and L2 to prevent catalysis. (**I**) HNH domain of structure in (**G**) positioned by linkers L1 and L2 after complete TS-sgRNA R-loop formation of ~17 bp. L1 residues – 765–780. L2 residues – 906–918. (**J**) SpCas9 HNH cuts the TS, and RuvC cuts NTS to produce blunt-ended DNA. Active site residues (not including Mg^2+^): RuvC – Asp10, Glu762, His982, His983, Asp986. HNH – Asp837, Asp839, His840, Asn863. PAM, protospacer adjacent motif; PI, PAM-interacting. NTS, non-target strand, TS, target strand. REC, recognition.

Type II CRISPR-Cas9 effector complexes have emerged as exceptional biotechnological tools for a variety of DNA manipulations both *in vitro* and *in vivo* [23]. Cas9 proteins have evolved to function in their bacterial hosts with a two-component gRNA composed of a CRISPR RNA (crRNA) and a *trans*-activating CRISPR RNA (tracrRNA) that can be fused into a tracrRNA:crRNA chimera called a single-guide RNA (sgRNA) [10,24]. It is the combination of its programmability and exquisite conformational control that underpins Cas9 as a successful biotechnological tool. *Streptococcus pyogenes* (Sp) Cas9 with sgRNA will be the model system for this discussion about allostery, where we explore the conformational checkpoints towards nuclease activation (Figure 1) [25].

The first conformational checkpoint for any CRISPR-Cas effector to surpass is gRNA (or sgRNA) binding. Apo-SpCas9 shows a bilobed architecture consisting of a ‘recognition’ or ‘REC’ lobe (REC1-3) and the ‘nuclease’ or ‘NUC’ lobe (HNH, RuvC and protospacer adjacent motif (PAM)-interacting (PI)) (Figure 1A). In solution, apo-SpCas9 is highly dynamic, but upon sgRNA binding, is accompanied by a major conformational shift in REC1-3 to wrap around the sgRNA (Figure 1B) [26,27]. Prior to target DNA binding, both apo and sgRNA-loaded SpCas9 adopt a conformation where the two nuclease domains, RuvC and HNH, are autoinhibited in a conformation that limits DNA cleavage activity [27–29] (Figure 1C). Armed with an ~100 nt sgRNA, SpCas9 screens genomic DNA in a process called ‘target search’. Accessing the sequence information buried within double-stranded DNA requires that the DNA strands be separated. The key conformational rearrangements to separate the DNA involve the recognition of an NGG sequence known as the PAM that occurs directly 3′ to the target [10,30]. PAM recognition is a conformational checkpoint and is critical to both the speed of Cas9 activity and its fidelity in gene editing [31]. Using the PI domain, SpCas9 scans the major groove of dsDNA in search of an NGG sequence in the non-target strand (NTS) (i.e. the DNA strand that does not base pair with sgRNA, as opposed to target strand (TS)) (Figure 1D) [32–34]. SpCas9 has two PI arginine residues that confer NGG PAM selectivity (Figure 1E), while diverse Cas9 homologs select alternative PAM sequences using alternative PI residues [35]. PAM-containing DNA is released much slower compared with PAM-lacking DNA, providing a kinetic window to hold the DNA in place for the intrinsic conformational dynamics of the REC lobe to occur [32]. Critically, REC2 pivots towards the bound DNA and uses several lysine residues to contact the phosphate backbone (Figure 1F) [36]. The combination of these stabilising interactions allows time for intrinsic SpCas9 motions to bend the DNA and partially pry open its target sequence [36,37]. Using this PAM recognition conformational checkpoint, SpCas9 both selects for potential DNA targets and efficiently pries apart DNA strands.

With a partially unwound DNA, SpCas9 interrogates whether the bound PAM-containing DNA contains the correct target sequence. Partially melted DNA targets are first probed by a set of five bases within the sgRNA known as the seed (Figure 1E) [10,33]. Correct base pairing between the TS and sgRNA seed is a strict requirement for SpCas9 activity. Mismatches result in DNA dissociation and the SpCas9–sgRNA complex being recycled to restart target search [37]. Complete seed-target base pairing drives further DNA unwinding from the PAM-proximal to distal regions [37]. Complete target hybridisation to the sgRNA is another conformational checkpoint named ‘R-loop formation’, that is, the formation of a stable three-stranded nucleic acid structure [38]. The propagating sgRNA-target DNA heteroduplex drives a conformational change in the REC domains to accommodate the R-loop (Figure 1G) [38–40].

To precisely cleave an identified DNA target, the HNH nuclease of SpCas9 must translocate to the scissile phosphate after sufficient base pairing has occurred. With only ~9 bp of complementarity between the sgRNA and DNA TS, the R-loop is incomplete and the HNH nuclease is stalled from translocating [41–43] (Figure 1H). Approximately 16 bp is required to achieve the HNH shift [41–45], and the REC domains are critical in detecting this activation threshold [38]. Crossing the ~16 bp threshold elongates the target-sgRNA heteroduplex (along with ssDNA from the NTS) to trigger the HNH adopting an active state [26,46,47]. Here, the HNH nuclease pivots towards the TS by flexible linkers (L1 and L2) driven by allosteric changes in the REC domains (Figure 1I). REC2 has an inverse conformational relationship with the HNH, where an HNH shift towards the cut site is accompanied by a concerted REC2 shift away from the cut site [48,49]. REC1, meanwhile, functions in locking the HNH nuclease in position after completing the transition to aid TS cleavage. The required shifts in the L1 and L2 linkers to achieve the HNH shift transduce an allosteric signal to activate the RuvC [50]. Having reached the active conformation, SpCas9 carries out concerted cleavage of both the TS and NTS of the bound DNA (Figure 1J) [26,43,46,47]. Supporting this model is the fact that active site mutations in either the HNH or RuvC will produce a Cas9-nickase that only cleaves a single DNA strand [10,51]. After cleavage, dsDNA remains stably associated in the SpCas9 R-loop effectively rendering Cas9 a single turnover enzyme [52,53].

The conformational checkpoints directing SpCas9 also apply to evolutionarily related systems. Divergent orthologs, such as the *Neisseria meningitidis* (Nme) Cas9, exhibit similar conformational controls through PAM recognition, seed binding, R-loop formation and HNH/RuvC nuclease activation [54]. More recently described, the ancestral IscB/IsrB and Cas9-D relatives appear to share similar conformational checkpoints [55,56]. For example, the more compact Cas9-D systems exhibit large conformational shifts in the REC lobe during target hybridisation similar to SpCas9 [57,58]. In contrast, the IscB/IsrB proteins, while containing a NUC lobe with HNH and RuvC, have a REC lobe composed of a non-coding ωRNA that functions to sense target DNA hybridisation and gate HNH translocation-induced RuvC activation [59]. These recent structural studies highlight that the conformational control of Cas9 likely evolved as an early evolutionary solution to control nuclease activation.

## Timing the activity of CRISPR-Cas12a

The discovery of type V Cas12 systems heralded an exciting expansion of the CRISPR-Cas toolbox beyond Cas9 [60,61]. We focus our discussion on the prototypical subtype Cas12a (formerly Cpf1) and a representative *Francisella novicida* Cas12a (FnCas12a), effectors defined by the presence of a single RuvC nuclease domain and a short ~40 nt gRNA [61]. While Cas12a is evolutionarily unrelated to Cas9 [62], it exhibits many conformationally and allosterically similar checkpoints to control gRNA loading, target DNA recognition and activation of the RuvC nuclease [63,64].

**Figure 2:**
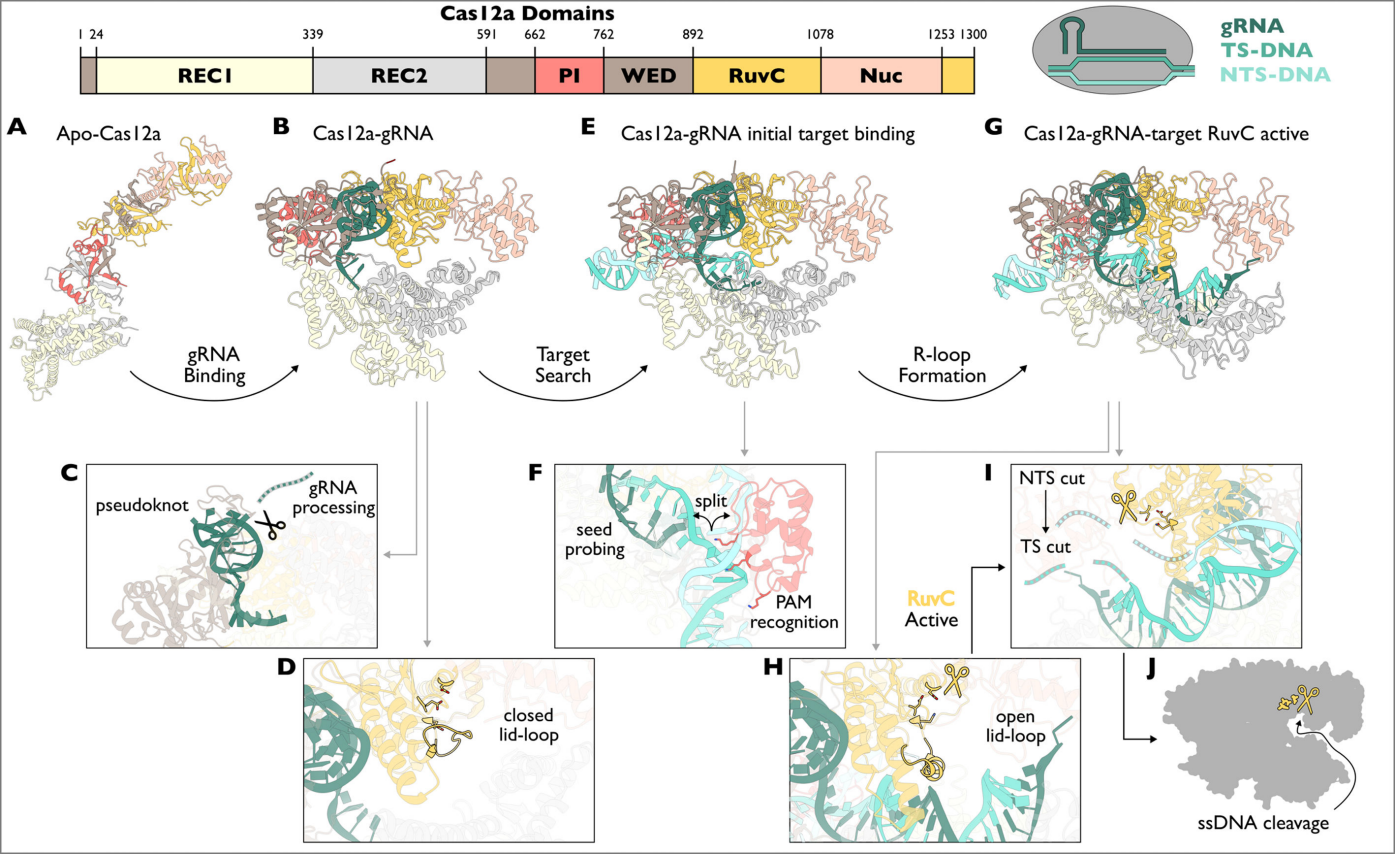
Conformational checkpoints and molecular mechanisms of *Francisella novicida* Cas12a. RuvC active site residues: Asp917, Glu1006 and Asp1255. Lid-loop residues: 1008–1021. (**A**) Apo-FnCas12a in elongated conformation. Positively charged surfaces on WED and REC domains recruit gRNA for binding. (PDB: 8H9D). (**B**) FnCas12a-gRNA, where gRNA binding stabilises the compact conformation of FnCas12a. (PDB: 5NG6). (**C**) gRNA repeat has a pseudoknot that is bound by the WED and RuvC domains. A nuclease domain cleaves pre-gRNA where it exits the complex at the 5′ end. (**D**) RuvC is inactived by lid-loop conformation covering active site residues. (**E**) FnCas12a-gRNA-target binding PAM and beginning to associate with gRNA. (PDB: 6GTC). (**F**) PAM-interacting lysine residues hold target in place for DNA unwinding to occur and for base pairs to form between gRNA seed and target. This structure exhibits pre-base paired conformation of target. PAM recognition of lysine residues – Lys667, Lys671 and Lys677. (**G**) FnCas12a-gRNA-target with full target complementarity. REC domains shift to accommodate gRNA-target R-loop. (PDB: 6GTG). (**H**) The Lid-loop over RuvC active site is opened to switch on catalytic activity. (**I**) FnCas12a cleaves NTS then TS to produce DNA with a 5′ overhang. (**J**) FnCas12a-gRNA-target complex in post-cleavage state. The RuvC nuclease remains active and solvent exposed to facilitate non-specific ssDNA cleavage. (PDB: 5MGA).

Like all class 2 CRISPR-Cas systems, Cas12a is bilobed with a REC and NUC lobe (Figure 2). The REC lobe is made up of domains REC1 and REC2, while the NUC lobe is composed of the wedge (WED), PI, RuvC and Nuc domains. In its apo-state, these lobes of Cas12a display large rotations at the REC2-WED junction to adopt an elongated (Figure 2A) or compact configuration [65,66]. The dynamics between elongated and compact forms are thought to expose the positively charged guide binding pocket to the solvent, where it recruits and entrenches the gRNA inside the compact form [67] (Figure 2B). Inside the Cas12a-gRNA complex, the 5′ direct repeat of the Cas12a gRNA has a distinctive pseudoknot structure bound through a combination of sequence and structure-specific recognition by the WED and RuvC domains [68]. After binding the gRNA, Cas12a carries out a catalytic precursor gRNA (pre-gRNA) processing reaction that removes nucleotides from the gRNA 5′-end beyond the pseudoknot, thus creating mature gRNA (Figure 2C) [69]. This step is tightly controlled by the conformation of the system where the conserved WED domain RNase active site forms around the bound pre-gRNA 5′ end [70,71]. Having bound and processed gRNA, mature Cas12a-gRNA effectors are then competent to search for target dsDNA via a PAM-mediated mechanism.

Without a target, Cas12a suppresses nuclease activity using a discrete structure known as the lid-loop to cover the RuvC active site (Figure 2D). Uncovering the active site requires the correct complementary DNA target to base pair with gRNA [72]. Like SpCas9, Cas12a conformationally controls the process of DNA unwinding and R-loop formation through a PAM recognition checkpoint [61,68,73,74]. PAM-lacking dsDNA is rapidly rejected, while those with the correct PAM (typically T-rich) are recognised by conserved lysine residues in the PI domain that is optimally oriented after gRNA binding [75,76]. Following PAM-binding (Figure 2E), the PI domain becomes significantly less flexible to support subsequent DNA unwinding. The PI lysine residues play an important role in holding the PAM. Of note, Lys671 (FnCas12a) is inserted into the target duplex, distorting the DNA strands sufficiently to begin the unwinding process (Figure 2F) [74]. Stable association with a PAM-containing dsDNA facilitates DNA distortion and exposes the target DNA to the gRNA for base pairing (Figure 2F) [73–75,77], akin to SpCas9 [36].

Following PAM recognition and DNA distortion, the gRNA seed probes the target strand for further complementarity [73,74]. Importantly, most of the gRNA spacer is disordered in the complex except for the first 5–8 nt of the spacer known as the seed region, which is directly adjacent to the direct repeat [71,78]. The seed here is pre-ordered in an A-form helix to favour base pairing with the TS nucleotides. Stable base pairing between the gRNA seed and TS DNA allows for an extended R-loop to propagate and bring about conformational changes in the REC1 and REC2 domains (Figure 2G) [73,74,77]. The target DNA is only stably bound to the complex after an ~17 bp R-loop has formed [78,79]. Simultaneously, Cas12a transitions into a state with an active RuvC nuclease domain, effectively coupling stable target DNA binding as a checkpoint to nuclease activation [75,80]. The resulting duplex induces major conformational changes in the PAM-distal end of the complex with REC1 and REC2 having the most dramatic change. In addition, the overall stability of the complex is increased conferred by a combination of new secondary structures that interact with the newly formed duplex [81]. For example, the bridge-helix, which connects different lobes of the protein, now features an extended helix from a more flexible state [82]. This change, along with rearrangements in critical REC-linkers and RuvC structural elements drives the lid-loop away from the RuvC nuclease to allow target DNA access and cleavage (Figure 2H) [72,77,81].

The active Cas12a effector complex utilises a large degree of conformational flexibility to enable both TS and NTS cleavage by the same RuvC active site. Two PAM-distal domains, Nuc and REC2, have increased conformational flexibility once a target has bound. The active RuvC first cleaves the NTS and then the TS [71]. Following NTS cleavage, TS cleavage is facilitated by the intrinsic flexibility of the PAM-distal duplex R-loop junction, which allows access to the RuvC nuclease (Figure 2I) [78–80]. The result is that Cas12a makes staggered cuts to the target DNA with a ~4 nt overhang at the 5′ end [83,84].

With limited interactions holding it in place, the PAM-distal DNA is released from the Cas12a effector after cleavage. However, the RuvC remains active. In this post-cleavage state, Cas12a may cleave freely diffusing ssDNA or, to a lesser extent, RNA (Figure 2J) [85,86]. This cleavage of nucleic acids that are not bound to the effector is coined as cleavage in *trans* (or *trans*-cleavage) as opposed to cleavage in *cis* of a bound target. The target-specific activation of Cas12a as a constitutive nuclease has been leveraged for DNA detection methods such as DETECTR [87]. Here, *trans*-cleavage of substrate ssDNA brings about a signal (typically fluorescence) that can only occur if the correct target is available in the sample [85]. Of note, Cas12a has also been made into a high-fidelity tool for gene editing applications [88,89]. *Trans-*cleavage would be expected to be detrimental to gene editing outcomes since it would introduce less controlled nuclease activity. However, it has not been reported to interfere with gene editing experiments thus far [90] and has no detectable role in bacterial immunity [91]. This could be explained by gRNA displacing R-loops [81] or by the relative inaccessibility of ssDNA in cellular contexts.

## Conformational control in type VI CRISPR-Cas13a

The conformational checkpoints governing activity of RNA-targeting CRISPR-Cas13a systems (formerly C2c2) have critical differences from the DNA-targeting Cas9 and Cas12a systems [48]. Cas13a has gRNA that base pairs with target ssRNA to activate an RNase active site formed between two higher eukaryotic and prokaryotic nucleotide-binding (HEPN) domains [92,93]. Once activated, Cas13a cleaves solvent-accessible segments of the target RNA (*cis*-cleavage) and accessible off-target ssRNA (*trans*-cleavage) [92,93]. This characteristic of Cas13a stands in stark contrast with the mechanism of DNA-targeting Cas9 and Cas12a systems and is facilitated by an enzyme structure that distances target RNA binding domains from the activated nuclease [94].

**Figure 3:**
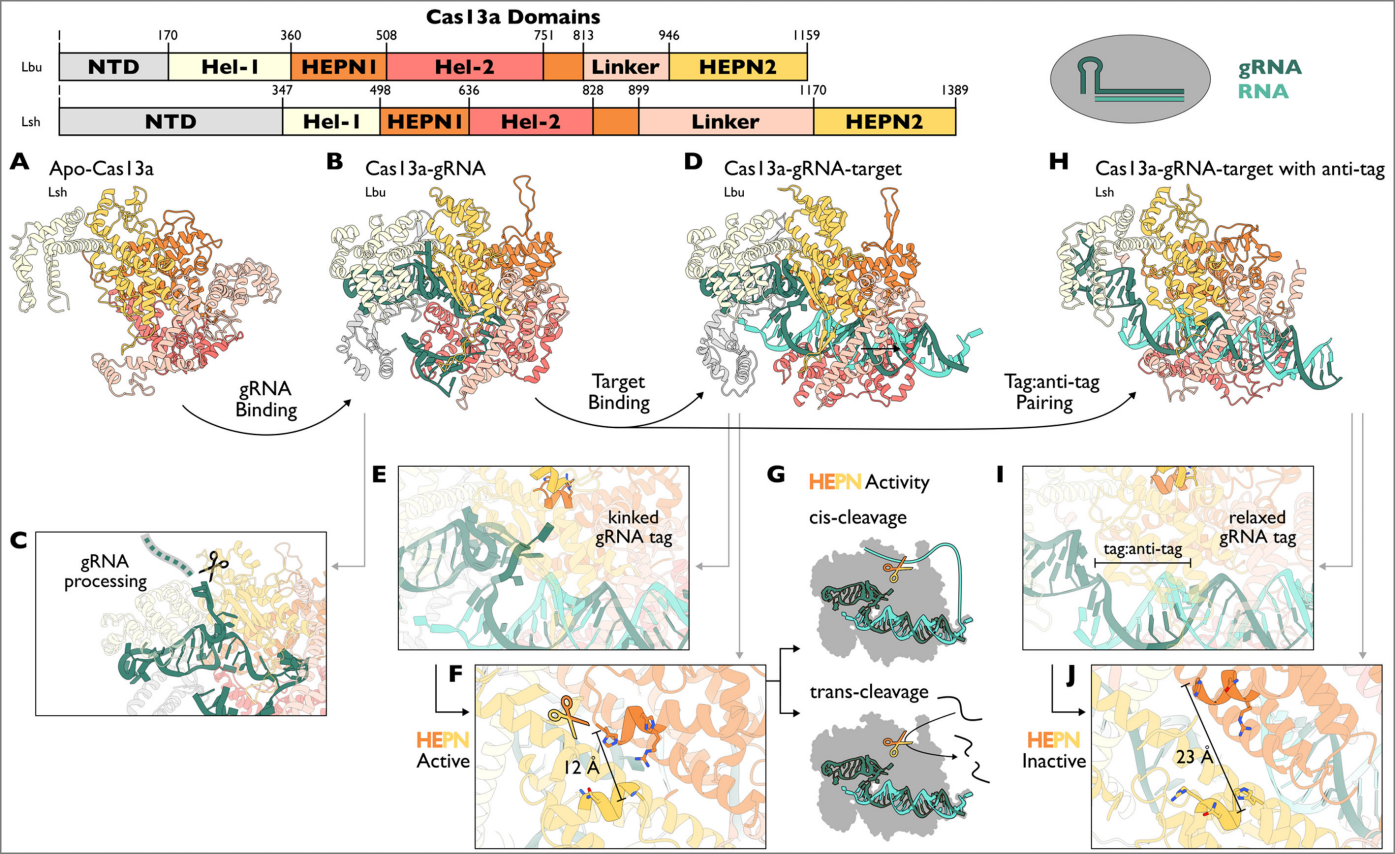
Conformational checkpoints and molecular mechanisms of *Leptotrichia shahii* and *Leptotrichia buccalis* Cas13a. (**A**) Apo-LshCas13a with gRNA binding pocket exposed to solvent. (PDB: 5WTJ) (**B**) LbuCas13a-gRNA ready for target binding. (PDB: 5XWY). (**C**) A nuclease domain cleaves pre-gRNA where it exits the complex at the 5′ end. (**D**) LbuCas13a-gRNA-target complex with an activated HEPN. (PDB: 5XWP). (**E**) HEPN is allosterically activated by interactions with gRNA tag (in the direct repeat) in a kinked conformation. (**F**) Close proximity of HEPN1 and HEPN2 catalytic residues facilitates nuclease activity. LbuCas13a HEPN active site residues: Arg472, His473, His477, Arg1048, Asn1049 and His1053. Residues 1048 and 1053 have been mutated to Ala. (**G**) Schematic of active LbuCas13a-gRNA-target cleaving RNA in *cis* and *trans* using the same HEPN active site. (**H**) LshCas13a-gRNA-target with tag:anti-tag pairing. (PDB: 7MDQ). (**I**) Target containing an anti-tag binds gRNA spacer and the tag. This relaxes the gRNA kink as shown in (**E**). (**J**) Tag: anti-tag pairing prevents HEPN catalytic residues from coming into close proximity. LshCas13a HEPN active site residues: Arg597, Asn598, His602, Arg1278, Asn1279 and His1283.

Cas13a is described as a bilobed effector protein with a REC lobe consisting of the N-terminal domain (NTD) and helical-1 while the NUC lobe contains HEPN1, HEPN2, Helical-2 and a linker domain (Figure 3). In its apo state, Cas13a exhibits an open pre-gRNA binding pocket in the REC lobe between NTD, helical-1 and HEPN2 domains (Figure 3A) [94]. Cas13a, like Cas12a, binds to and matures pre-gRNA by catalysing an RNA cleavage reaction near the 5′ end [94–99]. The pre-gRNA repeat adopts a compact stem-loop structure that is bound by Cas13a using a combination of sequence- and structure-specific contacts mediated by the helical-1 and HEPN2 domains (Figure 3B). The resulting complex forms a composite pre-gRNA processing nuclease at the interface between each domain that drives processing of the pre-gRNA through cleavage of the proximal scissile phosphate (Figure 3C) [94–97]. The final product is a mature Cas13a-gRNA effector complex that is competent for target ssRNA recognition.

To bind a target RNA and activate its HEPN nuclease, Cas13a first probes target RNA using the seed of its gRNA. The seed is composed of gRNA nucleotides that are solvent-exposed and function as the first point of contact with a potential target RNA [97]. As such, mismatches within the seed significantly attenuate the chance of successful Cas13a activation [100]. Upon seed binding, gRNA and target begin to duplex which, when complete, drives Cas13a to form an activated HEPN nuclease (Figure 3D). In contrast with Cas9 and Cas12a, the Cas13a gRNA itself makes the largest conformational change to become an ordered duplex, which is stabilised by many contacts to the protein [97]. Cas13a interacts with the target RNA backbone, including direct contacts with the 2′-OH, which explains the strong preference to target RNA over DNA [92,93,97]. The shape of the duplex is also strictly monitored. Notably, Cas13a scans a ‘switch region’ in the duplex that requires perfect complementarity before HEPN nuclease activation can occur [100,101]. Furthermore, gRNA nucleotides in the direct repeat immediately adjacent to the spacer are reorganised upon duplex formation to form a sharp kink (Figure 3E) [97]. This gRNA shape is a crucial signal for allosteric HEPN activation, resulting in a rearrangement that brings the conserved catalytic residues from HEPN1 and HEPN2 together [101] (Figure 3F).

The activated Cas13a HEPN nuclease is exposed to solvent and far from the gRNA-target duplex in the complex’s interior. Target RNA can be cleaved by Cas13a in *cis* if it is long enough to extend beyond the duplex and reach the active site [92,93] (Figure 3G). In contrast with Cas12a, which required target cleavage before ssDNA *trans-*cleavage could occur, the Cas13a HEPN supports bystander RNA cleavage in *trans* immediately following HEPN nuclease activation (Figure 3G). Activated HEPN nucleases in Cas13a have a preference for cleaving both target and bystander RNA at U or A nucleotides, depending on the homolog [95]. Furthermore, recent reports suggest that Cas13a preferentially cleaves tRNA anticodons, implying that the HEPN nuclease has an as yet uncharacterised substrate selection process [102]. Nevertheless, the core catalytic mechanism underpinning *cis* and *trans* cleavage is believed to be identical.

As targets are ssRNA, their exposed nucleotides that do not base pair with the gRNA have the potential to make detrimental interactions for Cas13a activity. This has been demonstrated with ssRNA targets that have further complementarity to gRNA beyond the spacer and into the direct repeat, in what is called ‘tag: anti-tag pairing’ (where gRNA has the ‘tag’ and the target has the ‘anti-tag’) (Figure 3H). If this occurs, the RNA duplex between the gRNA and target protrudes into the gRNA binding pocket and relaxes the sharp kink that promotes HEPN activation (Figure 3I) [103,104]. In some Cas13a, extra gRNA-target complementarity by even a single base pair may significantly attenuate the catalytic output of the HEPN nuclease [92,105]. These targets fail (or at least are less capable) to bring the HEPN catalytic residues into alignment, thereby suppressing RNA cleavage (Figure 3J) [104]. These observations highlight that there is an optimum Cas13a structure to align catalytic HEPN residues, exemplifying its stringent conformational regulation.

## The evolutionary relevance of CRISPR-Cas conformational control

CRISPR-Cas effectors are adapted for the co-evolutionary arms race between microbes and phage. Microbial life, at risk of lethal infection, mounts defences to neutralise infection from phage, which in turn strive to evade such defences [106]. Every microbial immune system operates by sensing a molecular phage signature before an immune response can be brought about to eliminate the threat of infection. Currently, there is an increasing search for immune systems with novel mechanisms of detection and/or interference [107–112]. By and large, microbial immune systems tend to be innate, the most common of which are restriction enzymes that identify sequences of nucleic acid by direct protein-nucleotide contacts [113–116]. In contrast, CRISPR-Cas systems recognise that a phage infection has occurred through gRNA sequence-specific contacts with phage nucleic acid and switch conformation based on the shape of a gRNA-target duplex. Fidelity of activation, which is important to prevent lethal self-targeting, is ensured by sensing for gRNA-target mismatches that manifest as irregularities in an otherwise perfect duplex [117,118]. Coupled to a series of elaborate regulatory conformational checkpoints, this is an evolutionarily successful immune sensory and interference strategy. Phage populations frequently change the targeted regions of their genomes in response to CRISPR-Cas [119–121]. The stringent activation of CRISPR-Cas might be regarded as a disadvantage because it implies CRISPR-Cas defence may be easily evaded by phage via negligibly costly mutations. However, no CRISPR-Cas effector complex can perfectly distinguish the ‘true’ target from every similar sequence – there is often a degree of tolerance. This tolerance must not be so relaxed that it is harmful to the host (by targeting its own genome), but it provides a critical advantage to recognise mutating phage escapers. In fact, some bacteria maintain immunity through other processes that acquire gRNA against new phage populations [122–125], which can be enhanced when gRNA-target pairing is imperfect [126,127]. This emphasises the potent selection pressures on CRISPR-Cas to be adaptable to changing targets but without compromising on the specificity garnered through conformational control.

**Figure 4:**
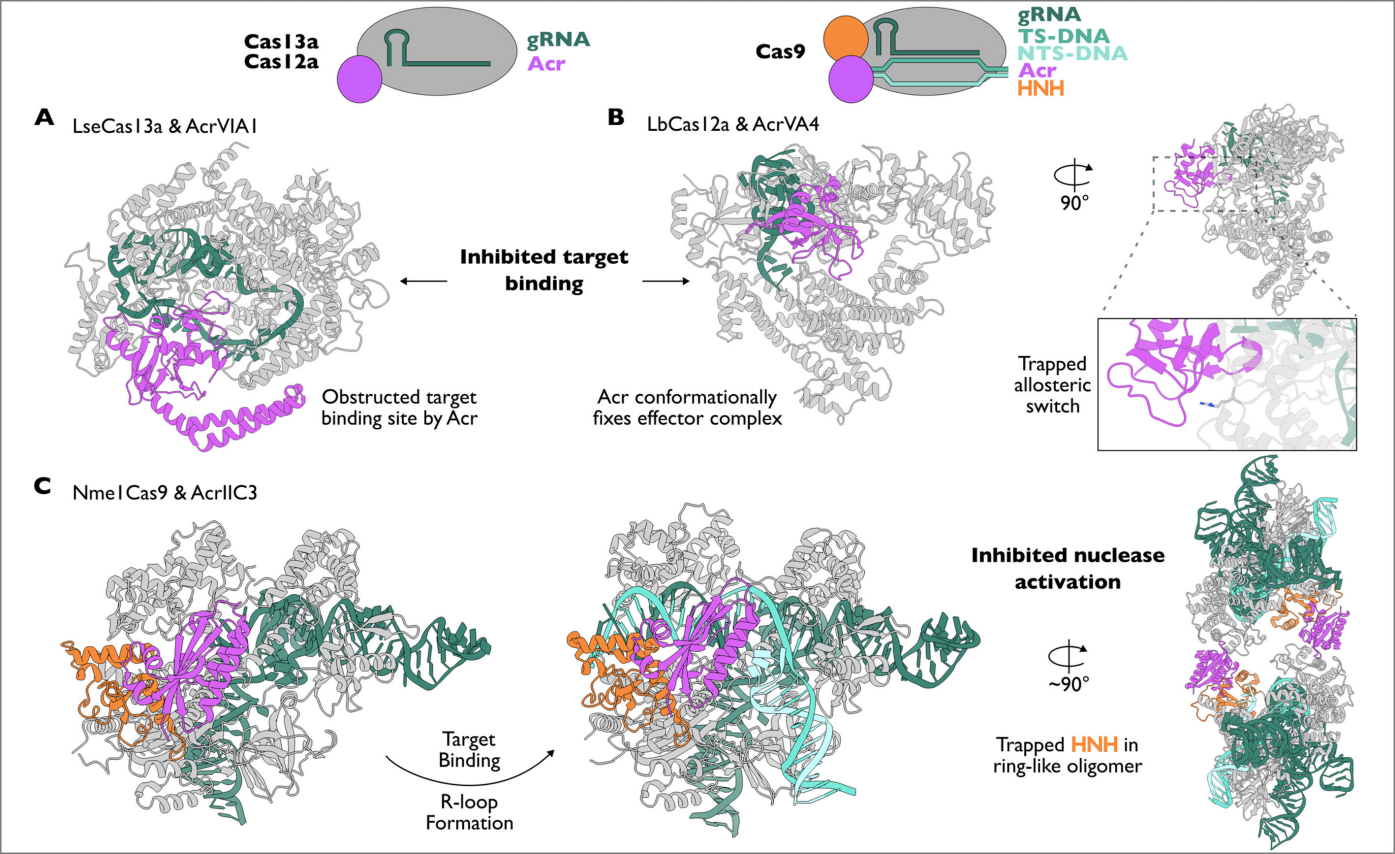
Mechanisms of inhibition by anti-CRISPRs targeting Cas13a, Cas12a and Cas9. (**A**) Target binding for *Listeria seeligeri* Cas13a-gRNA is inhibited by AcrVIA1 by blocking the surface that target RNA is first recognised to prevent duplex formation. (PDB: 6VRB) (**B**) Target binding for *Lachnospiraceae* bacterium Cas12a-gRNA is inhibited by AcrVA4 by binding to allosteric switch Arg887, which must rotate towards the interior of the complex to facilitate gRNA-target duplex formation. AcrVA4 locks Arg887 in the inactive conformation to prevent duplex formation. (PDB: 6P7M) (**C**) HNH conformational shift for *Neisseria meningitidis* Cas9-sgRNA is inhibited by AcrIIC3 despite duplex formation occurring. 2 copies of NmeCas9-sgRNA-AcrIIC3 (PDB: 6JE9) and NmeCas9-sgRNA-target-AcrIIC3 (PDB: 6JE4) are co-ordinated in a ring-like formation. NmeCas9 HNH domain residues – 541–655.

On the opposing side of the arms race, phages have acquired tools to actively inhibit CRISPR-Cas defence by exploiting their requirement for conformational checkpoints [128–130]. The incredible diversity of CRISPR-Cas systems is reflected in the variety of anti-CRISPRs (Acrs) that are specific to certain CRISPR systems. To date, there are numerous examples of Acrs that block key conformational transitions in CRISPR-Cas9, Cas12a and Cas13a complexes underscoring that this is an evolutionarily successful mechanism of phage resistance (Figure 4). For example, AcrVIA1 and AcrVA4, which act on *Listeria seeligeri* (Lse) Cas13a and *Lachnospiraceae bacterium* (Lb) Cas12a, respectively, inhibit target binding to gRNA [98,131]. AcrVIA1 binds directly to gRNA at the solvent-exposed seed of the LseCas13a-gRNA complex, essentially competing with target RNA for binding and effectively blocking the commitment to nuclease activation (Figure 4A) [98]. Conversely, AcrVA4 binds LbCas12a at an interface that is distant from the target binding architecture. Rather than competitive inhibition, this Acr prevents R-loop formation with DNA targets by caging a bridge-helix arginine residue to prevent a conformational switch within LbCas12a (Figure 4B) [131,132]. Finally, AcrIIC2 acts on NmeCas9 but does not prevent binding to the target DNA [54]. Rather, it prohibits target cleavage by securing the flexible HNH domain in a pre-catalytic state (before it is translocated to the scissile phosphate). To achieve this, two AcrIIC2 proteins work together to bind each HNH and REC2 domain from two NmeCas9 complexes in a ring-shaped formation (Figure 4C) [54]. Interestingly, other Acrs for NmeCas9 demonstrate effective inhibition to multiple Cas9 homologs. These more broad-spectrum Acrs occur because they bind a highly conserved interface within the HNH domain [133,134]. These limited examples speak to the breadth of Acrs in nature and the success that comes with targeting conformational checkpoints in highly diverse CRISPR-Cas immune systems [13]. For a more detailed description of Acr evolution and mechanism of action, we refer the reader to several excellent reviews [135–139].

## Implications of conformational control in biotechnology

CRISPR-Cas systems have proven invaluable in recent years for the development of many novel applications to manipulate nucleic acids [16]. Conformational checkpoints and the control of transitions between inactive and active states are an important aspect of CRISPR-Cas application in biotechnology. CRISPR-Cas systems function on a complex conformational energy landscape with active Cas protein-gRNA-target complexes only achieved after overcoming complex thermodynamic and kinetic barriers [140,141]. Since Cas9, Cas12a and Cas13a effector complexes do not consume ATP, the conformational barriers to overcome should be thermodynamically favourable (or at least energetically inexpensive). Any binding event inherently comes with some entropic cost, so it must be accompanied by energetic gains to compensate, either by stabilising interactions (enthalpic) or increasing flexibility (entropic). For example, gRNA-binding is a thermodynamically favourable transition for effector complexes as investigated through *in silico* studies [142,143]. In agreement, thermal melting assays find that gRNA-bound states have increased thermal stability [67,144,145]. This rationale similarly explains how gRNA-target hybridisation induces conformational changes in the Cas effector protein. By forming new base pairing and stacking interactions, the system is provided with sufficient energy to overcome barriers and transition to the nuclease active state. This conformational landscape of CRISPR-Cas complexes should be considered as biotechnologies continue to emerge, including conformational traps, pitfalls and bottlenecks. We refer the reader to recent reviews about protein folding as the theoretical foundation of this discussion [146,147].

The reaction speeds of CRISPR-Cas nucleases are in many cases bottlenecked by the time it takes to find complementary targets. This is because an effector complex must locate a target in a complex mixture of other cellular material, which may interact or even bind periodically with the complex. Having mechanisms to quickly reject interacting molecules significantly enhances the overall kinetics of target search. For example, Cas9 efficiently searches for DNA targets in a complex mixture of genomic DNA, which has proven effective in the context of genome editing [148–150]. When Cas9 productively encounters DNA, it quickly scans to determine whether a PAM is present relative to the time needed to resolve subsequent steps of seed binding, R-loop formation and HNH translocation [37,151]. Essentially, long-lived conformational transitions in Cas9 are only committed to after brief screening for a PAM. Therefore, PAMs facilitate timely completion of DNA-targeting applications. Reflecting this fact is that near PAMless Cas9 constructs exhibit poor genome editing efficiencies because they are less able to reject non-targets [152,153]. The one advantage of using constructs with less stringent PAMs is that they increase the range of targetable sites but at the cost of editing kinetics. Rather than changing the NGG to N-ything, a more applicable solution to provide more genome coverage is to use a repertoire of Cas proteins with distinct PAMs [31].

Cas12a and Cas9 are fundamentally different enzymes, not just in terms of their evolution, structure or function, but also with distinct advantages and disadvantages afforded by their unique conformational regulation. Cas12a enzymes have a T-rich PAM in contrast with G-rich Cas9 PAM (e.g. TTTV is the PAM of FnCas12a [154]), a property that already expands the accessible targeting space. While optimised Cas12a and SpCas9 have similar editing efficacy, Cas12a may induce deletions at a higher rate and of larger size [88,155]. It has been hypothesised this occurs because the PAM-distal regions of the target DNA are cleaved, preserving the PAM-proximal seed after non-homologous end joining (NHEJ). Furthermore, the cleavage site of Cas12a has some slight plasticity given that Cas12a relies on flexibility around the R-loop to cleave the target [79,156]. Altogether, multiple rounds of target cleavage and repair may occur until the target-binding sequence has >5 nt deletions. SpCas9-mediated editing tends to bring about less deletions, since it cleaves the target proximal to the seed, which is the most important region for target binding [157,158]. Therefore, SpCas9 is more sensitive to mutations introduced by NHEJ than Cas12a. However, Cas12a can be easily co-opted for multiplexed editing across different genomic loci given its ability to autonomously process pre-gRNA into many individual gRNA [159,160]. A major limitation of SpCas9 and its larger sgRNA that can be difficult to fit inside delivery vectors [161,162]. Altogether, this emphasises the advantage of having a range of well-characterised CRISPR systems for use in biotechnology because a larger toolbox enables more diverse procedures.

In some cases, it can be advantageous to slow the overall speed of a CRISPR-Cas reaction to suppress detrimental reactions. A common issue with genome editing is off-target cleavage, that is, activation of the nuclease in response to binding to a DNA sequence that resembles the target. Considering the therapeutic potential for genome editing, limiting the extent of off-target cleavage is a high priority to mitigate adverse effects. gRNA-target mismatches tend to be more tolerated at positions where the duplex does not make direct contacts with the Cas protein, resulting in a blind spot [39]. High-fidelity CRISPR-Cas constructs have been generated to address this. For SpCas9, several constructs have been designed [163,164] that hamper the translocation of the HNH catalytic domain [46]. In effect, the energy threshold to activate the HNH has increased and can only be overcome with a correct target. The result is a Cas9 that favours off-target dissociation and disproportionately reduces the rate of off-target cleavage relative to cleaving the correct target [165].

Reaction kinetics must be carefully considered for Cas13a given that *trans*-cleavage activity is multi-turnover. Cas12a *trans*-cleavage activity for ssDNA is not detectable during phage defence [91,166] and has not been reported to occur in several gene editing studies [88,155,167,168]. Similarly, Cas13a has demonstrated the capability to specifically knock down RNA via *cis*-cleavage in spite of its known *trans*-cleavage activity [169]. When expressed in mammalian cells, Cas13a is reported to exhibit either highly specific RNA knockdown or widespread RNA degradation [169–171]. These conflicting outcomes suggest an equilibrium between *cis* and *trans*-cleavage that is highly dependent on the homolog, cell type, expression levels and other variables [172,173]. An engineered construct of Cas13d (closely related to Cas13a) that exhibits reduced *trans*-cleavage activity relative to *cis*-cleavage was recently reported with mutated residues proximal to the HEPN nuclease [174]. While the structural mechanisms have yet to be established, it was recently demonstrated that Cas13d specificity is connected to its overall catalytic efficiency [175]. Consistent with this, engineered Cas13a constructs that enhance *trans*-cleavage utilise an RNA-sequestering mechanism, essentially increasing the local concentration of bystander RNA around the activated HEPN nuclease [176].

Diagnostic methods to detect DNA or RNA from patient samples have been developed relying on the *trans*-cleavage activity of Cas12a or Cas13a, respectively [85,177]. Here, binding of the target initiates widespread *trans*-cleavage of reporter ssDNA or RNA that fluoresces upon cleavage [95]. The limiting factor for many of these procedures is the concentration of target, which in clinically relevant contexts can reach attomolar scale, because there is abundance of Cas protein-gRNA complex in reaction mixtures [178]. This limitation ensures only a small population of effectors becomes activated, which may be insufficient to produce a detectable signal. Moreover, some gRNAs have spacers that are less prone to activate nuclease activity [179–181]. Using a variety of spacers combined with a target amplification step may be used to account for these but at a cost of time and expense. Another innovation is to couple *trans*-cleavage with the activation of supplementary nucleases [182]. Fundamentally, their purpose is to increase the rate of reporter cleavage because doing so increases the limit of detection and decreases reaction time.

## Conclusions and prospects for CRISPR-Cas design

Conformational checkpoints can be thought of in terms of conditional ‘if X then Y’ statements [183]. For CRISPR-Cas nucleases, this translates to ‘if the correct target is bound then activate the nuclease’. As we have described, this is achieved in practice through discrete conformational checkpoints that ultimately gate activation of the nuclease domain. A comprehensive understanding of conformational control and its complexity will become increasingly valuable as more sophisticated methods of protein discovery and engineering are explored. In recent years, computational protein design and structure prediction have rapidly improved with artificial intelligence (AI) [184,185]. AI has also enabled the development of computational protein design tools such as RoseTTAFold diffusion [186,187]. While these approaches have limitations [188–192], the development of next-generation CRISPR-Cas tools may stand to gain from the integration of AI and our understanding of conformational checkpoints. For example, conformational checkpoints are missing from CRISPR-Cas and effector domain fusions, including base editors [193,194] and prime editors [195]. Each component (CRISPR-Cas and the fused effector domain) is connected via a flexible linker but uncoordinated, like two balls on a string. Future efforts could theoretically include a feedback mechanism where a target nucleic acid is bound and drives activation of the base or prime editor [196,197]. Furthermore, current best-in-class CRISPR-Cas effectors derived from nature may soon be replaced by synthetic, computationally determined constructs using deep-learning language models that retain knowledge of the conformational checkpoints [198,199]. Recently, a fully synthetic complex named OpenCRISPR-1 was generated by AI to compete with SpCas9 as the *de facto* gene editor in the field [200]. OpenCRISPR-1 is predicted to have a very similar structure to that of SpCas9 and therefore similar conformational regulations despite the limited sequence identity. We anticipate more synthetic CRISPR-Cas effectors to be produced in the years to come with a myriad of desirable characteristics. For instance, generally small CRISPR-Cas enzymes are convenient to work with in a gene editing context due to the size limitations imposed by delivery vectors [201]. Furthermore, SpCas9 mutants with significant deletions remain comparably fast to wildtype, indicating that a large portion of its protein sequence could be discarded while maintaining enzymatic competence [202]. Protein engineering is fundamentally a combinatorial problem with any amino acid having 20 possible side-chain identities. Deep-language learning models have the capacity to navigate the vast sequence space, and novel CRISPR-Cas enzymes may be sampled by setting appropriate constraints to maintain conformational control [203]. Ultimately, what computational protein engineering can and cannot do is still being explored with simple *de novo* designed proteins showing promise for future work [204]. CRISPR-Cas systems will be good models with elaborate conformational controls to test the limits of these new avenues for molecular engineering.

Despite significant attention over the years, there are many CRISPR-Cas systems whose function have not been explored. Microbial immune systems are under pressure to innovate, lest they become countered by phage [205,206]. In the evolutionary arms race between microbes and phage, it is certain that CRISPR-Cas has evolved and is continuing to evolve a diverse repertoire of defence mechanisms. Computational tools are becoming increasingly necessary to explore these novel CRISPR-Cas systems with unique mechanisms of action and conformational regulations. The amount of metagenomic data has significantly increased thanks to advancements in high-throughput sequencing, and in turn, so has the number of identified CRISPR-Cas systems [207]. It is now possible to develop sequence to structure pipelines to view novel CRISPR-Cas systems and screen for interesting candidates such as those with additional catalytic or binding domains. This represents a significant turning point in the field surrounding CRISPR-Cas. Initially, CRISPR-Cas was discovered by curiosity-driven studies into repetitive elements of microbial genomes. Later, the mechanisms and structures of CRISPR-Cas effectors were characterised. In future, researchers may be using the predicted structural information about CRISPR-Cas effectors to guide further discovery and ‘direct the cut’ in novel applications.

**Perspectives**

- Macromolecular conformational change is often an essential regulatory mechanism to finely control biochemical activity. CRISPR-Cas complexes exhibit whole conformational networks with discrete checkpoints gating nuclease activation.
- As adaptive immune systems, CRISPR-Cas evolution is highly constrained to impose conformational switches that limit host self-destruction.
- The development of next-generation CRISPR-Cas tools will require significant computational input to account for the intrinsic dynamics and conformational switches that are crucial for these systems to function.

## Abbreviations

AI: artificial intelligence
CRISPR: clustered regularly interspaced short palindromic repeats
Cas: CRISPR-associated
HEPN: higher eukaryotic and prokaryotic nucleotide-binding
NHEJ: non-homologous end joining
NTD: N-terminal domain
NTS: non-target strand
PAM: protospacer adjacent motif
PI: PAM-interacting
REC: recognition
crRNA: CRISPR RNA
gRNA: guide RNA
pre-gRNA: precursor gRNA
sgRNA: single-guide RNA
tracrRNA: *trans*-activating CRISPR RNA
